# Incidence and Presenting Characteristics of Angiosarcoma in the US, 2001-2020

**DOI:** 10.1001/jamanetworkopen.2024.6235

**Published:** 2024-04-12

**Authors:** Michael J. Wagner, Vinod Ravi, Stephanie K. Schaub, Ed Y. Kim, Jeremy Sharib, Harveshp Mogal, Min Park, Michaela Tsai, Daniela Duarte-Bateman, Anthony Tufaro, Elizabeth T. Loggers, Lee D. Cranmer, Bonny Chau, Michael J. Hassett, Juneko Grilley-Olson, Kelly G. Paulson

**Affiliations:** 1 Clinical Research Division, Fred Hutchinson Cancer Center, Seattle, Washington; 2 Division of Medical Oncology, University of Washington, Seattle; 3 Department of Medical Oncology, Dana-Farber Cancer Institute, Harvard University, Boston, Massachusetts; 4 Department of Sarcoma Medical Oncology, MD Anderson Cancer Center, Houston, Texas; 5 Department of Radiation Oncology, University of Washington, Seattle; 6 Department of Surgery, University of Washington, Seattle; 7 Department of Medical Oncology, Providence-Swedish Cancer Institute, Seattle, Washington; 8 Department of Plastic and Reconstructive Surgery, Cleveland Clinic, Cleveland, Ohio; 9 Duke Cancer Institute, Duke University Medical Center, Durham, North Carolina

## Abstract

**Importance:**

Angiosarcoma is an aggressive vascular malignant neoplasm presenting either as a primary or secondary cancer, often arising after radiotherapy or in the context of preexisting lymphedema. Comprehensive data describing its incidence and presentation patterns are needed.

**Objective:**

To describe the incidence, presenting characteristics, and change over time of angiosarcoma in the US.

**Design, Setting, and Participants:**

This retrospective cross-sectional study used data from the US Cancer Statistics (USCS) National Program of Cancer Registries–Surveillance, Epidemiology, and End Results Combined Database, which captures more than 99% of newly diagnosed cancers in the US. The study included all 19 289 patients in the US with a new diagnosis of angiosarcoma between 2001 and 2020 captured in the USCS database. Statistical analysis was performed from June to September 2023.

**Main Outcomes and Measures:**

Incidence of angiosarcoma, demographics of patients with angiosarcoma, and extent of disease at presentation.

**Results:**

The study included 19 289 patients (median age, 71 years [IQR, 59-80 years]; 10 506 women [54.5%]) with a new diagnosis of angiosarcoma. The US incidence of angiosarcoma doubled between 2001 (657 cases) and 2019 (1312 cases), reflecting both an increase in the adjusted incidence rate of 1.6% per year (*P* = .001), to 3.3 cases per 1 000 000 person-years (95% CI, 3.1-3.5 cases per 1 000 000 person-years), and an increase in the population at risk. In 2020, the reported incidence rate (3.0 cases per 1 000 000 person-years) and cases of angiosarcoma (n = 1159) were modestly lower than in 2019. Overall, 72.3% of cases of angiosarcoma (n = 13 955) were cutaneous, subcutaneous, or breast angiosarcomas; 24.4% were visceral (n = 4701); and 3.3% were located in unknown or rare primary sites (n = 633). Secondary breast and chest wall angiosarcomas among women represented the largest contribution to increasing incidence. Among breast angiosarcomas, 99.2% (2684 of 2705) were in women and 71.9% (1944 of 2705) were secondary. A total of 80.4% of chest wall or thorax cases among women (1861 of 2316) were secondary vs 26.5% among men (112 of 422), and 63.9% of upper extremity cases among women (205 of 321) were secondary vs 26.8% (56 of 209) among men (*P* = .001). Rates of secondary angiosarcoma in the abdomen and lower extremities were similar between men and women. The incidence rate of visceral angiosarcoma was also found to be increasing (1.5% per year; *P* = .001).

**Conclusions and Relevance:**

This cross-sectional study describes angiosarcoma presentation patterns and incidence rates in the US over a 20-year period and shows that the number of cases in men and women increased, with the greatest increase among women with secondary angiosarcoma of the chest, breast, and upper extremity. These data increase awareness of a rare but highly morbid disease and highlight the need for improved early detection of angiosarcoma among patients at high risk, such as women with a history of breast cancer.

## Key Points

**Question:**

What are the incidence, presenting characteristics, and change in incidence over time of angiosarcoma in the US?

**Findings:**

This cross-sectional study of 19 289 patients with a new diagnosis of angiosarcoma found that the incidence of angiosarcoma is increasing among men and women in the US, with more than 1000 new cases diagnosed per year. This overall increase is associated with a higher incidence rate of secondary breast and chest wall angiosarcomas among women.

**Meaning:**

These data increase awareness of a rare but highly morbid disease and highlight the need for monitoring and increased suspicion of angiosarcoma among patients at higher risk, such as women who have a prior history of breast cancer.

## Teaser

This cross-sectional study uses data from the US Cancer Statistics National Program of Cancer Registries–Surveillance, Epidemiology, and End Results Combined Database to describe the incidence, presenting characteristics, and change over time of angiosarcoma in the US.

## Introduction

Angiosarcoma is an aggressive cancer of endothelial cells that can arise in any part of the body but occurs most commonly in the skin.^1^ It is now recognized that angiosarcoma subtypes can be characterized by differing molecular signatures. Angiosarcomas that arise secondary to radiation or chronic lymphedema (Stewart-Treves syndrome^2^) are characterized by *MYC* amplification,^3^ whereas primary angiosarcomas of the breast are more likely to harbor *PI3K* mutations.^4^ Cutaneous angiosarcomas of the scalp or face are more likely to harbor a DNA mutation pattern consistent with UV light damage and a high tumor mutational burden, suggesting responsiveness to immune checkpoint inhibitors.^4,5^ Recent insights into angiosarcoma biology have led to renewed efforts to target the molecular underpinnings of angiosarcoma through multicenter clinical trials.^6,7^

Despite this progress, the true incidence of angiosarcoma remains uncertain. A French registry study estimated the incidence of angiosarcoma to be 0.15 per 100 000 person-years but noted that the incidence between 2007 and 2016 was 0.26 per 100 000 person-years, higher than in the preceding decades.^8^ A US-based registry study found a similar rate during that time period and also identified an increasing incidence over time specifically among older patients, most notably those older than 75 years.^9^

Given the strong association of breast cancer treatment with the rare but serious later development of angiosarcoma, a previous study investigated the incidence of breast angiosarcoma in the US using the Surveillance, Epidemiology, and End Results (SEER) database across all ages and identified an increasing incidence of angiosarcoma among patients previously treated for breast cancer.^10^ Large retrospective series have identified other factors associated with risk of angiosarcoma development among patients with breast cancer, including hypertension and diabetes, in addition to the previously recognized risk of prior receipt of radiotherapy.^11,12^ We sought to identify trends for angiosarcoma in the US population and to better describe the observed increased risk over time.

## Methods

### Population and Data

This cross-sectional study was approved by the Providence institutional review board and followed the Strengthening the Reporting of Observational Studies in Epidemiology (STROBE) reporting guideline.^13^ A retrospective cross-sectional database analysis was conducted using the US Cancer Statistics (USCS) National Program of Cancer Registries–SEER Combined Database, which captures more than 99% of newly diagnosed cancers in the US.^14^ All US patients with a new diagnosis of angiosarcoma (*International Classification of Diseases for Oncology, 3rd Edition,* codes 9120/3: hemangiosarcoma, malignant) between 2001 and 2020 (n = 19 289) were included. To differentiate primary vs secondary angiosarcomas, we separated patients for whom angiosarcoma was the first diagnosis of a malignant neoplasm and those who had a history of other malignant neoplasms before the angiosarcoma diagnosis per the USCS. The latter cases were considered secondary. Due to the limitations of the database, we could not determine with certainty whether an angiosarcoma in a patient with a history of malignant neoplasms was secondary to prior cancer treatment. Case numbers fewer than 16 were suppressed to comply with privacy requirements. The main outcomes were incidence of angiosarcoma, patient demographics, including history of cancer before angiosarcoma diagnosis, and extent of disease at presentation. Race and ethnicity were determined using the “Race and origin recode” variable sourced by the North American Association of Central Cancer Registries.

### Statistical Analysis

Statistical analysis was performed from June to September 2023. For incidence rate calculations, data were age adjusted to the 2000 US standard population (19 age groups; census P25-1130), and 95% CIs were estimated using the methods of Tiwari et al.^15^ Two-year mean values were used to calculate incidence trends, with the annual percentage change (APC) calculated by the weighted least-squares method. Angiosarcoma subgroups of interest included cutaneous, breast, and visceral angiosarcoma, with further breakdown by primary site. SEER*Stat software, version 8.4.1.2 (National Cancer Institute) was used to extract data.^16^ Analyses were performed within SEER*Stat. Graphs were made in GraphPad Prism, version 9 (Dotmatics). All *P* values were from 2-sided tests and results were deemed statistically significant at *P* < .05.

## Results

### All Angiosarcoma

**Figure 1.**
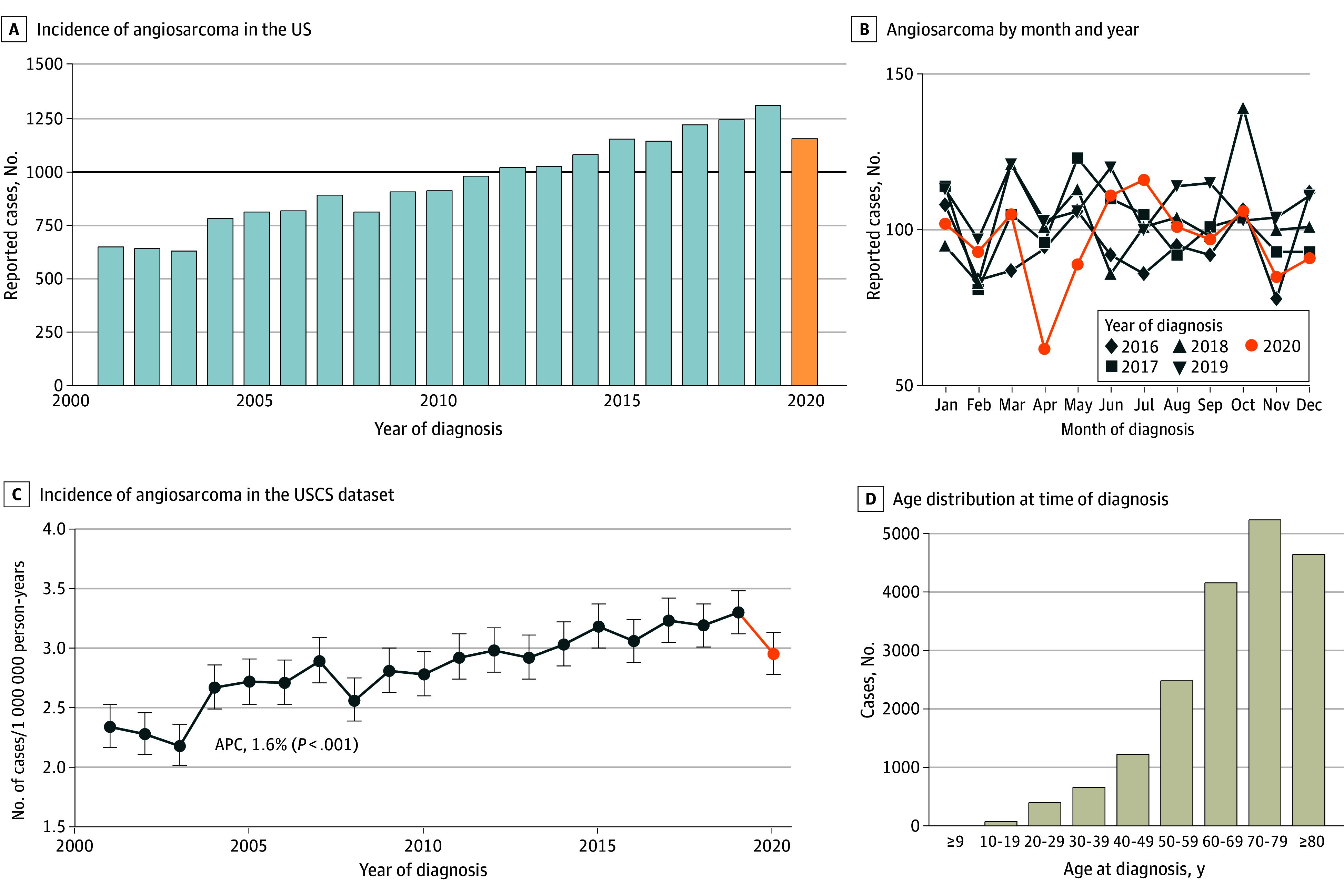
Incidence of Angiosarcoma in the United States (N = 19 289) A, Cases per year of incident (newly diagnosed) angiosarcoma in the US; the black horizontal line indicates 1000 newly diagnosed cases per year, and the orange bar indicates year 2020, when reported incidence was impacted by COVID-19 pandemic. B, Reported cases of angiosarcoma by month and year, 2016-2020. C, Incidence rate of angiosarcoma in the US Cancer Statistics (USCS) dataset, adjusted for population age and sex. Error bars indicate 95% CIs. D, Age distribution at time of diagnosis. APC indicates annual percentage change.

Angiosarcoma was reported in 19 289 patients (median age, 71 years [IQR, 59-80 years]; 10 506 women [54.5%]) (Figure 1D). Overall, 72.3% of cases (n = 13 955) were cutaneous, subcutaneous, or breast angiosarcomas; 24.4% (n = 4701) were visceral angiosarcomas; and 3.3% (n = 633) were located in unknown or rare primary sites (eTable and eFigure 1 in Supplement 1). A total of 14 967 patients (79.9%) were non-Hispanic White, 1622 patients (8.7%) were Black, and 1405 patients (7.5%) were Hispanic. The US incidence of angiosarcoma doubled between 2001 and 2019 from 657 cases to 1312 cases (Figure 1A). This increase corresponded to an increase in the population-adjusted incidence rate of 1.6% per year (*P* = .001), to a current rate of 3.3 cases per 1 000 000 person-years (95% CI, 3.1-3.5 cases per 1 000 000 person-years) (Figure 1C) and an increase in the population at risk. A total of 8679 of 16 067 patients (54.0%) presented with localized disease, 2649 of 16 067 (16.5%) with regional disease by extension, 753 of 16 067 (4.7%) with nodal involvement, and 3932 of 16 067 (24.5%) with distant metastatic disease (eFigure 1D in Supplement 1).

### Year 2020

In 2020, the reported incidence of angiosarcoma was reduced by 11.7% (1159 cases vs 1312 cases in 2019; rate of 3.0 cases per 1 000 000 person-years [95% CI, 2.8-3.1 cases per 1 000 000 person-years] compared with 3.3 cases per 1 000 000 person-years [95% CI, 3.1-3.5 cases per 1 000 000 person-years] in 2019). Essentially all this reduced incidence was accounted for by the months of April and May 2020 (Figure 1B).

### Cutaneous and Breast Angiosarcomas

**Figure 2.**
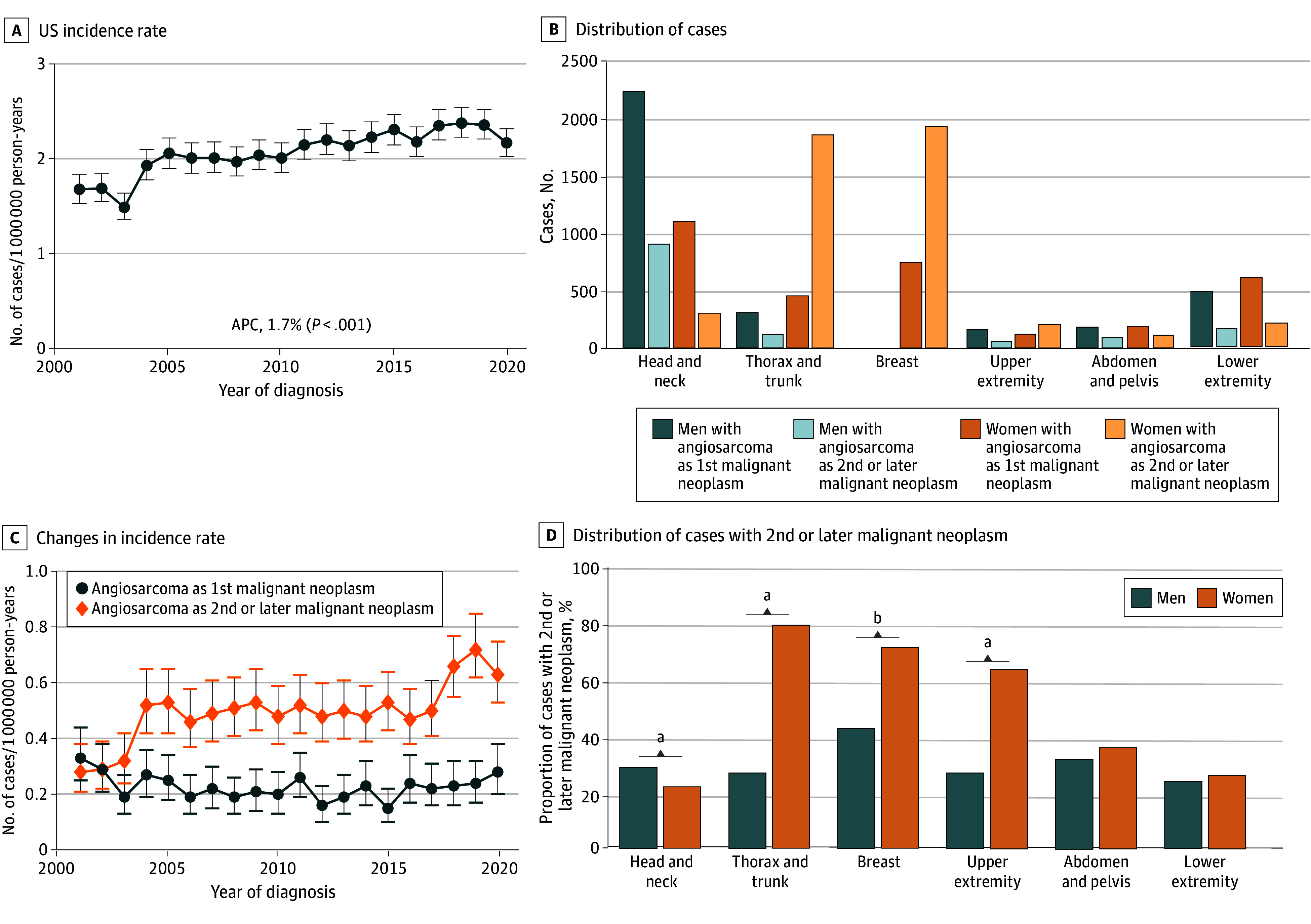
Cutaneous, Subcutaneous, and Breast Angiosarcoma (N = 13 955) A, US-reported incidence rate, 2001-2020. Error bars indicate 95% CIs. B, Breakdown by body site, sex, and first or second or later malignant neoplasm. C, Changes in incidence rate for breast angiosarcoma among women based on whether the angiosarcoma was the first reported malignant neoplasm or second or later reported malignant neoplasm. Error bars indicate 95% CIs. D, Percentage by site and sex that are the second or later malignant neoplasm for the patient. APC indicates annual percentage change. ^a^*P* < .001. ^b^*P* = .006.

The incidence rate of all skin, subcutaneous, and breast angiosarcomas increased 1.7% per year over the study period to 2.3 cases per 1 000 000 person-years (95% CI, 2.2-2.5 cases per 1 000 000 person-years; *P* = .001) (Figure 2A), corresponding with an increase in cases from 464 in 2001 to 939 in 2019. The median age at diagnosis of all skin, subcutaneous, and breast angiosarcomas was 72 years (IQR, 62-80 years) (eFigure 2 in Supplement 1); 11 210 of 13 586 patients (82.5%) were non-Hispanic White, and 8484 of 13 955 patients (60.8%) were female. There were significant differences in the sites of diagnosis between men (67.0% head and neck [3148 of 4700]; 9.0% thorax or trunk [422 of 4700]; 0.4% breast [21 of 4700]; 5.7% abdomen [268 of 4700]; 4.4% upper extremity [209 of 4700]; and 13.4% lower extremity [632 of 4700]) and women (18.0% head and neck [1412 of 7844]; 29.5% thorax or trunk [2316 of 7844]; 34.2% breast [2684 of 7844]; 3.8% abdomen [298 of 7844]; 4.1% upper extremity [321 of 7844]; and 10.4% lower extremity [813 of 7844]) (*P* < .001). There were marked differences between the proportion of men (28.1% [1320 of 4699]) and the proportion of women (58.9% [4622 of 7844]) presenting with cutaneous or breast angiosarcoma as a secondary malignant neoplasm (*P* = .001). On further breakdown, the major differences included the thorax or trunk, the breast, and the upper extremity (Figure 2). Among breast angiosarcomas, 99.2% (2684 of 2705) were in women and 71.9% (1944 of 2705) were secondary. A total of 80.4% of chest wall or thorax cases among women (1861 of 2316) were secondary vs 26.5% among men (112 of 422), and 63.9% of upper extremity cases among women (205 of 321) were secondary vs 26.8% (56 of 209) among men (*P* = .001).

Rates of skin or soft-tissue angiosarcoma increased for both men and women; however, the sites of these increases were different between men and women. Among men, the rates of cutaneous and breast angiosarcomas were largely stable across sites from 2001 to 2020, with the exception of the head and neck; among men presenting with angiosarcoma of the head and neck as their first cancer, the incidence rate increased nonsignificantly by 0.9% per year (95% CI, 0.0%-1.8% per year) from 2001 to 2020 (*P* = .05), but the rate of angiosarcoma of the head and neck as a second or later cancer increased by 2.8% per year (95% CI, 1.4%-4.3% per year; *P* = .001). Similarly, among women, most of the increased incidence of angiosarcoma was accounted for by cases of breast angiosarcoma (APC, 2.7%; 95% CI, 1.5%-4.0%; *P* = .001) (Figure 2) occurring as a second or later malignant neoplasm, with no significant increases seen for the breast (−0.7%; 95% CI, −2.2% to 0.8%; *P* = .33) or other sites as the site of a first malignant neoplasm.

Among patients for whom their cutaneous, soft-tissue, or breast angiosarcoma was their first cancer diagnosed with known staging information, 58.6% (3767 of 6431) presented with localized disease, 18.6% (1198 of 6431) with regional disease, and 22.8% (1466 of 6431) with distant metastatic disease. For those presenting with angiosarcoma as their second or later cancer diagnosis, they were more likely to present with an earlier disease stage (*P* = .001): 6.5% local (3752 of 5641), 21.8% regional (1231 of 5641), and 11.7% distant metastatic (658 of 5641) (eFigure 3 in Supplement 1).

For women, the rate of breast cancer was stable over the period of study observation (APC, 0.1% per year; 95% CI, −0.1% to 0.3% per year) with an annual rate of 132 per 100 000 person-years in 2001 and 131 per 100 000 person-years in 2019 (there was a large decrease in 2020, to 119 per 100 000 person-years). For men, similar trends were observed (APC, 0.2% per year; 95% CI, −0.3% to 0.3% per year), although rates were much lower among men than among women (1.2 per 100 000 person-years in 2001 and 1.3 per 100 000 person-years in 2019).

### Visceral Angiosarcoma

**Figure 3.**
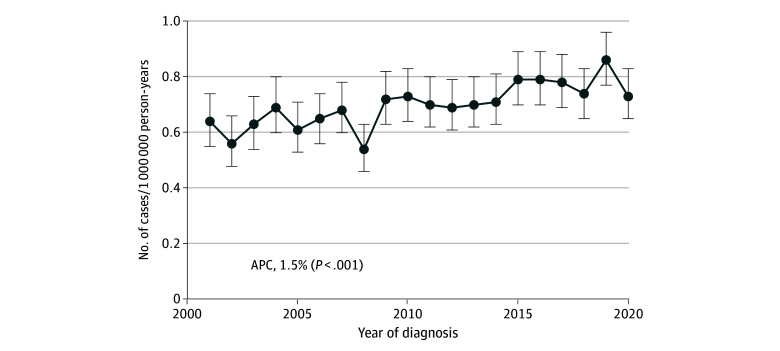
Incidence Rates of Angiosarcoma With Visceral Primary Sites Error bars indicate 95% CIs. APC indicates annual percentage change.

The reported incidence rate of visceral angiosarcoma is also increasing (1.5% per year [95% CI, 0.9%-2.1% per year]; *P* = .001) (Figure 3A) to a rate of 1 per 1 000 000 person-years, corresponding to approximately 300 to 330 cases per year in the US. The most common sites of visceral primary disease (n = 4701) were the liver (27.6% [n = 1299]), heart (13.4% [n = 632]), and bone (12.0% [n = 564]) (eFigure 4 in Supplement 1; Table). This distribution means that there are approximately 75 hepatic angiosarcoma cases, 45 cardiac and mediastinal cases, 35 bone cases, and 20 splenic cases annually. Persons presenting with angiosarcoma at visceral sites were, on average, younger (median age at diagnosis, 65 years [IQR, 52-75 years]; *P* = .001) than those presenting with skin, subcutaneous, or breast angiosarcoma (eFigure 2 in Supplement 1).

**Table.** Distribution of Primary Sites for Visceral Angiosarcoma

Site	No. (%) (N = 4701)
Head and neck	225 (4.8)
Esophagus and stomach	34 (0.7)
Small and large bowel	193 (4.1)
Hepatobiliary	1299 (27.6)
Pancreas	30 (0.6)
Trachea, lung, or pleura	431 (9.2)
Heart	632 (13.4)
Mediastinum	81 (1.7)
Bone	564 (12.0)
Spleen	334 (7.1)
Nerve	91 (1.9)
Retroperitoneum	193 (4.1)
Female reproductive system	102 (2.2)
Male reproductive system	46 (1.0)
Urinary	279 (5.9)
Endocrine	167 (3.6)

## Discussion

In this retrospective population registry–based cross-sectional study over a 20-year period, we show that the number of cases of angiosarcoma was increasing. The overall incidence was 3 per 1 000 000 person-years, which is slightly higher than the previously reported incidence in the US.^9^ The greatest increase was among women with secondary angiosarcoma of the chest, breast, or upper extremity. There was also an increase in incidence among men, especially those with angiosarcoma of the head or neck. This finding implies that prior cancer treatment (likely head or neck radiotherapy for men and breast cancer treatment for women) is likely associated with the marked increase in the observed incidence of cutaneous angiosarcoma. These data increase awareness of a rare but highly morbid disease and highlight the need for monitoring and increased suspicion of angiosarcoma among patients at higher risk, such as women who have a history of breast cancer.

Increased use of breast conservation therapy has enabled women to choose less-aggressive surgical interventions for their breast cancers. However, in rare cases, secondary sarcomas, including breast angiosarcoma, can develop. A previous study identified a trend of increasing incidence of secondary breast angiosarcoma while the incidence of primary breast angiosarcoma remained stable, and it showed that the percentage of patients receiving radiotherapy for breast carcinoma increased during this time.^10^ Here, we confirm that prior observation of increasing incidence of angiosarcoma and provide incidence estimates that can be used to aid in shared decision-making between patients with breast cancer and clinicians. The incidence of breast cancer during this period remained stable, so we can say that the increase in the incidence of angiosarcoma was not due solely to the increase in breast cancer incidence rates over that time period. Although radiotherapy is often no longer generally used for patients who receive a diagnosis of breast cancer after 65 years of age, approximately half of all breast cancers are diagnosed before 63 years of age.^17,18,19,20^ The risk of secondary angiosarcoma provides further impetus to not irradiate most people older than 65 years, given the lack of clinical benefit. There is also evidence that radiotherapy can be omitted for younger postmenopausal women at low risk for recurrence,^21,22^ and additional trials are ongoing to further evaluate the possibility of de-escalation of breast radiotherapy.^23^ Even though most patients with angiosarcoma present with localized disease, the extent of damage is often substantial, necessitating complex reconstructive surgery. Survival outcomes remain poor, which places considerable stress on patients, their families, and the health care system. By highlighting the increasing incidence of angiosarcoma specifically in the setting of women with a history of cancer, we hope to raise awareness of angiosarcoma as a rare but serious potential adverse effect so that cases can be identified early, thus increasing the chance for a long-term cure from both malignant neoplasms.

In addition to the increasing number of breast and chest wall angiosarcomas, we also found that the incidence of visceral angiosarcomas was increasing at the same rate as cutaneous angiosarcomas. The overall number of these cases is smaller than the number of cutaneous cases, but this trend is also intriguing and, to our knowledge, cannot be easily explained. One possibility is that some of these cases are also radiotherapy induced and are being caused by radiotherapy for cancers other than breast cancer. If this is the case, then newer radiotherapy techniques to minimize toxic effects may mitigate this risk over time.

Veiga et al^12^ identify hypertension and diabetes as significant risk factors for secondary soft tissue sarcomas, with relative risks of 4.8 and 5.3, respectively, compared with a relative risk of 8.1 for radiotherapy. These comorbidities may serve as confounders for our analysis. The age-adjusted incidence of diabetes among US adults has remained relatively stable between 2000 and 2021 but has increased among children and adolescents.^24^ Similarly, the age-adjusted prevalence of hypertension in the US remained largely stable from 1999 to 2018.^25^ Although we were not able to assess these comorbidities in our study population, monitoring and treating for diabetes and hypertension remain a cornerstone of preventive medicine and may play a role in reducing the risk of secondary sarcomas.

Just as soft tissue sarcomas are now recognized to be an increasingly diverse group of tumors defined by differing molecular features,^26^ angiosarcoma subtypes are increasingly becoming recognized by molecular alterations and clinical features. By lumping all angiosarcomas together in clinical trials, we risk missing critical positive signals from targeted agents or immunotherapy that may be highly effective for 1 subgroup. Therefore, one might consider designing a clinical trial with an attempt to capture as homogenous a group as possible by including only particular molecular subtypes of angiosarcoma or by preplanning statistical analysis by angiosarcoma subtypes. The epidemiologic data presented here can inform a trial design in this regard. The overall pattern of the molecular factors associated with angiosarcoma may be changing over time because there are more secondary cases. Awareness of this trend could one day affect treatment planning. Although angiosarcoma remains rare, the increasing incidence over the study period suggests that a sufficient number of cases may exist to accrue to a clinical trial with a more homogenous angiosarcoma population than has previously been attempted. The slight decrease in reported cases in 2020 likely represents reduced ascertainment secondary to COVID-19.^27^ As radiotherapy practices evolve with emerging evidence supporting the de-escalation of radiotherapy among selected patients with breast cancer, and with techniques such as brachytherapy or proton therapy that minimize radiation exposure to healthy tissue, it will be important to continue to assess the incidence of secondary sarcomas over time as recent trends may not predict future trends.

### Strengths and Limitations

This study has some strengths, including its use of a large comprehensive database that encompasses almost all cancer diagnoses in the US. Prior attempts to calculate the incidence of angiosarcoma were limited by small datasets precluding assessment of angiosarcomas by subtype.

This study also has some limitations, including the lack of data regarding the prior cancer diagnosis for cases in which a primary cancer was diagnosed before it would have been included in the USCS database. Similarly, although it is known that there is a lag between the time of first cancer diagnosis and a treatment-related angiosarcoma,^10,11,12^ we do not have data on time between malignant neoplasms for this entire cohort. Another limitation is that our data included only invasive breast cancer and thus excluded ductal carcinoma in situ, which is not uniformly captured in the national database. Because ductal carcinoma in situ is a noninvasive condition and not uniformly captured as a primary cancer, we suspect an underestimation of the number of secondary breast and chest wall angiosarcomas.

## Conclusions

This cross-sectional study found that the incidence of angiosarcoma is increasing in the US among men and women, with a notable increase seen among women previously treated for breast cancer. As molecular insights lead to a need for subtype-specific trials, with subgroups defined by anatomic or molecular characteristics, these epidemiologic data can inform clinical trial design moving forward for patients with angiosarcoma.

## Notes

Supplement 1. **eTable.** Demographics: All Patients With Available Data Included **eFigure 1.** Demographic Features of Persons Presenting With Angiosarcoma in the US **eFigure 2.** Age Distributions of Cutaneous and Visceral Angiosarcoma **eFigure 3.** Extent of Disease at Diagnosis for Skin/Soft Tissue Angiosarcoma Based on Whether First Malignancy Diagnosed or Second/Greater Malignancy Diagnosed for Patient **eFigure 4.** Angiosarcoma With Visceral Primary Sites: Distribution of Cases by Primary Site

Supplement 2.
Data Sharing Statement
