# Supplementary material for: Incidence and Presenting Characteristics of Angiosarcoma in the US, 2001-2020
Source: JAMA Netw Open. 2024 Apr 12;7(4):e246235. doi: 10.1001/jamanetworkopen.2024.6235 (PMC11015348; doi:10.1001/jamanetworkopen.2024.6235)
Supplement: Supplement 1. — eTable. Demographics: All Patients With Available Data Included eFigure 1. Demographic Features of Persons Presenting With Angiosarcoma in the US eFigure 2. Age Distributions of Cutaneous and Visceral Angiosarcoma eFigure 3. Extent of Disease at Diagnosis for Skin/Soft Tissue Angiosarcoma Based on Whether First Malignancy Diagnosed or Second/Greater Malignancy Diagnosed for Patient eFigure 4. Angiosarcoma With Visceral Primary Sites: Distribution of Cases by Primary Site [file jamanetwopen-e246235-s001.pdf]

Supplementary Online Content

Wagner MJ, Ravi V, Schaub SK, et al. Incidence and presenting characteristics of angiosarcoma in the US, 2001-2020. *JAMA Netw Open.* 2024;7(4):e246235. doi:10.1001/jamanetworkopen.2024.6235

eTable. Demographics: All Patients With Available Data Included
eFigure 1. Demographic Features of Persons Presenting With Angiosarcoma in the US
eFigure 2. Age Distributions of Cutaneous and Visceral Angiosarcoma
eFigure 3. Extent of Disease at Diagnosis for Skin/Soft Tissue Angiosarcoma Based on Whether First Malignancy Diagnosed or Second/Greater Malignancy Diagnosed for Patient
eFigure 4. Angiosarcoma With Visceral Primary Sites: Distribution of Cases by Primary Site

This supplementary material has been provided by the authors to give readers additional information about their work.

© 2024 Wagner MJ et al. *JAMA Network Open.*

eTable. Demographics: All Patients With Available Data Included

		Total	Percent
Age at dx (n=19289)	0-9 years	51	0.3
	10-19 years	111	0.6
	20-29 years	438	2.3
	30-39 years	698	3.6
	40-49 years	1267	6.6
	50-59 years	2529	13.1
	60-69 years	4209	21.8
	70-79 years	5289	27.4
	80+ years	4697	24.4
Sex (n=19289)	Male	8783	45.5
	Female	10506	54.5
Race/ethnicity (n=18731)	Non-Hispanic White	14967	79.9
	Black	1622	8.7
	American Indian/Alaska Native	93	0.5
	Asian or Pacific Islander	644	3.4
	Hispanic (All Races)	1405	7.5
Primary site (n=19289)	Skin/soft tissue	13955	72.3
	Visceral	4701	24.4
	Unknown, not reported or suppressed	633	3.3
LRD stage (n=16067)	Localized	8679	54.0
	Regional, direct	2649	16.5
	Regional, nodal	471	2.9
	Regional, direct & nodal	282	1.8
	Regional, NOS	54	0.3
	Distant	3932	24.5

© 2024 Wagner MJ et al. *JAMA Network Open.*

eFigure 1. Demographic Features of Persons Presenting With Angiosarcoma in the US

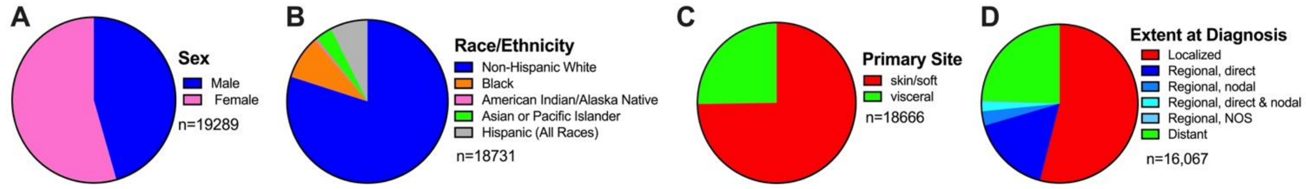

eFigure 1: Demographic Features of Persons Presenting with Angiosarcoma in the US. A) Sex distribution (n=19,289) **B)** Race/Ethnicity distribution (n=18,731) **C)** Primary site (skin/soft tissue versus visceral; n=18,666) **D)** LRD stage (n=16,067). This is a visualization of data contained in **eTable 1.**

© 2024 Wagner MJ et al. *JAMA Network Open.*

eFigure 2. Age Distributions of Cutaneous and Visceral Angiosarcoma

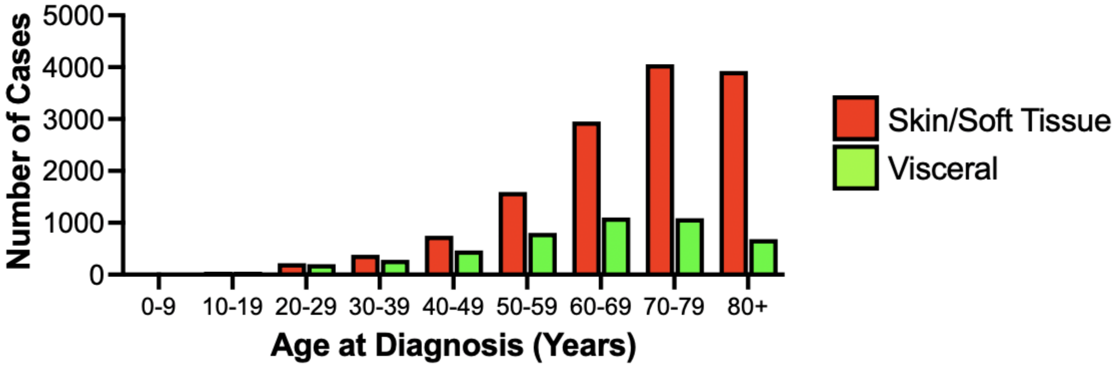

eFigure 2. Age Distributions of Cutaneous and Visceral Angiosarcoma. Age at diagnosis of angiosarcoma with skin/soft tissue primary (n=13955) and age at diagnosis of angiosarcoma with visceral primary (n=4697).

© 2024 Wagner MJ et al. *JAMA Network Open.*

eFigure 3. Extent of Disease at Diagnosis for Skin/Soft Tissue Angiosarcoma Based on Whether First Malignancy Diagnosed or Second/Greater Malignancy Diagnosed for Patient

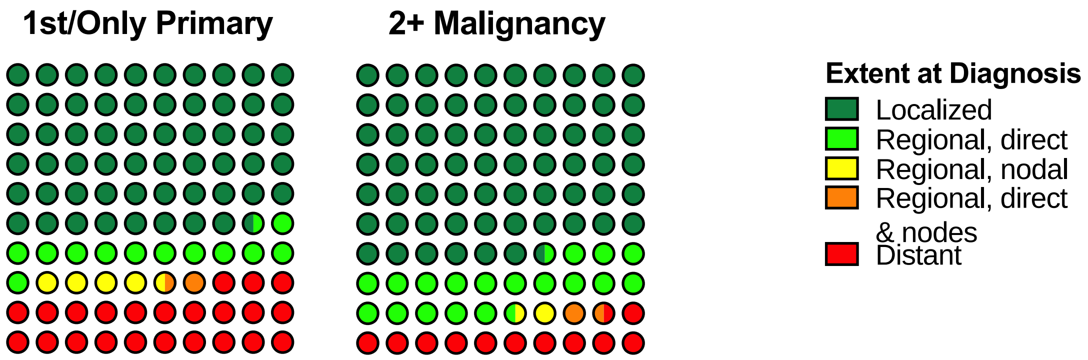

eFigure 3. Extent of disease at diagnosis for skin/soft tissue angiosarcoma based on whether first malignancy diagnosed or second/greater malignancy diagnosed for patient. N=6431 (first malignancy) and 5641 (second+ malignancy).

© 2024 Wagner MJ et al. *JAMA Network Open.*

eFigure 4. Angiosarcoma With Visceral Primary Sites: Distribution of Cases by Primary Site

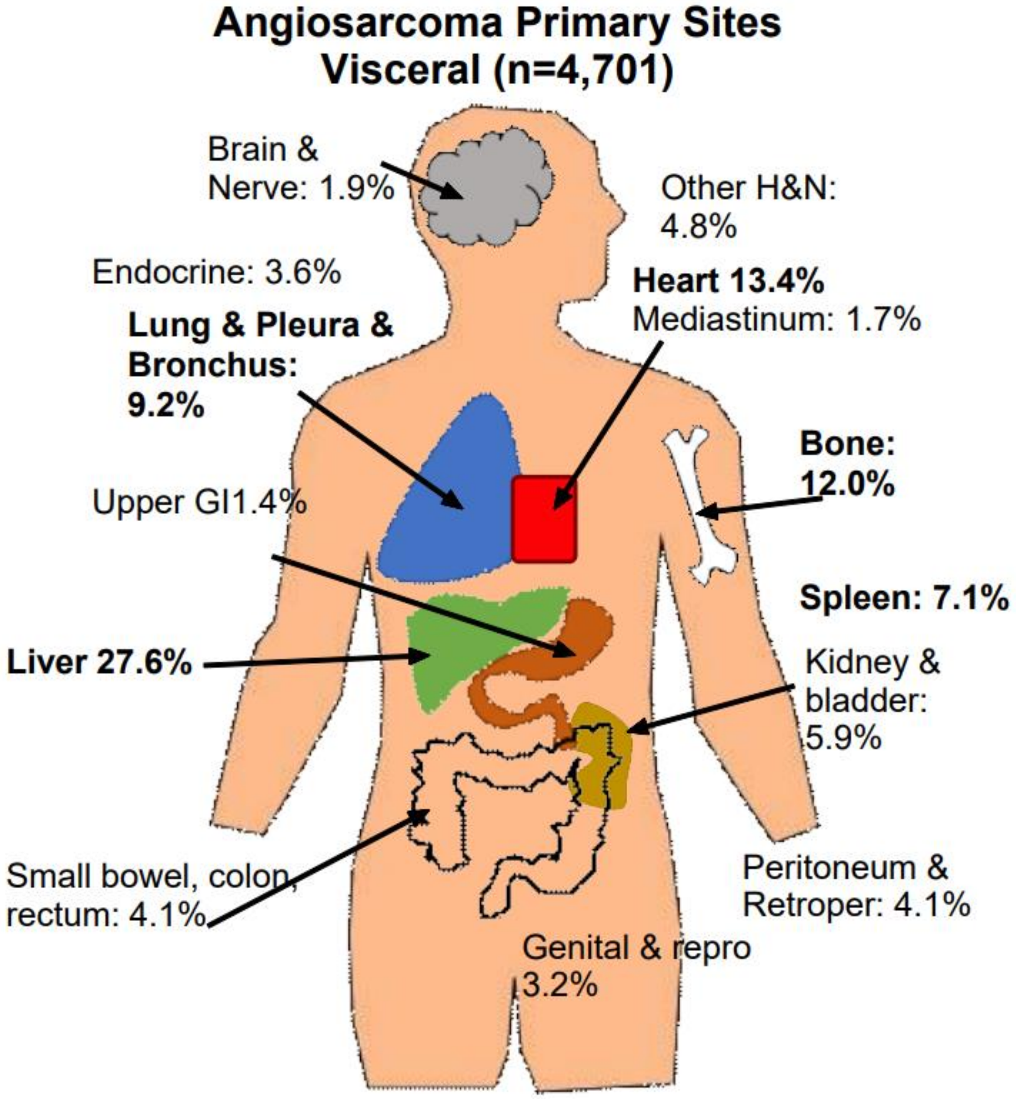

© 2024 Wagner MJ et al. *JAMA Network Open.*
