# Supplementary material for: Incidence and Presenting Characteristics of Angiosarcoma in the US, 2001-2020
Source: JAMA Netw Open. 2024 Apr 12;7(4):e246235. doi: 10.1001/jamanetworkopen.2024.6235 (PMC11015348; doi:10.1001/jamanetworkopen.2024.6235)
Supplement: Supplement 2. — Data Sharing Statement [file jamanetwopen-e246235-s002.pdf]

Data Sharing Statement

Wagner. Incidence and Presenting Characteristics of Angiosarcoma in the US, 2001-2020. *JAMA Netw Open*. Published April 12, 2024. doi:10.1001/jamanetworkopen.2024.6235

Data
Data available: Yes
Data types: Deidentified participant data, Other (please specify)
Additional Information: All data are publicly available through the U.S. Cancer Statistics (https://www.cdc.gov/cancer/uscs/index.htm)
How to access data: All data are publicly available through the U.S. Cancer Statistics (https://www.cdc.gov/cancer/uscs/index.htm)
When available: With publication

Supporting Documents
Document types: None

Additional Information
Who can access the data: All data are publicly available through the U.S. Cancer Statistics (https://www.cdc.gov/cancer/uscs/index.htm)
Types of analyses: Data are available for any purpose.
Mechanisms of data availability: All data are publicly available through the U.S. Cancer Statistics (https://www.cdc.gov/cancer/uscs/index.htm)
